# Bioinformatics Analysis Screening and Identification of Key Biomarkers and Drug Targets in Human Glioblastoma

**DOI:** 10.2174/0109298673316883240829073901

**Published:** 2024-09-06

**Authors:** Chunlei Wang, Ozal Beylerli, Yan Gu, Shancai Xu, Zhiyong Ji, Tatiana Ilyasova, Ilgiz Gareev, Vladimir Chekhonin

**Affiliations:** 1 Department of Neurosurgery, The First Affiliated Hospital of Harbin Medical University, Harbin, Heilongjiang Province, People’s Republic of China;; 2 Institute of Brain Science, Harbin Medical University, Harbin, Heilongjiang Province, People’s Republic of China;; 3 Central Research Laboratory, Bashkir State Medical University, Ufa, 450008, Russia;; 4 Department of Neurobiology, Pirogov Russian National Research Medical University of the Ministry of Healthcare of Russian Federation, Moscow, Russian Federation;; 5 Serbsky Federal Medical Research Centre of Psychiatry and Narcology of the Ministry of Healthcare of Russian Federation, Moscow, Russian Federation;; 6 The National Medical Research Center for Endocrinology, Moscow, Russian Federation

**Keywords:** Glioblastoma, bioinformatics analysis, diagnosis, therapeutic strategies, cancer progression, drugs

## Abstract

**Background:**

Glioblastoma is the most common type of brain cancer, with a prognosis that is unfortunately poor. Despite considerable progress in the field, the intricate molecular basis of this cancer remains elusive.

**Aim:**

The aim of this study was to identify genetic indicators of glioblastoma and reveal the processes behind its development.

**Objective:**

The advent and integration of supercomputing technology have led to a significant advancement in gene expression analysis platforms. Microarray analysis has gained recognition for its pivotal role in oncology, crucial for the molecular categorization of tumors, diagnosis, prognosis, stratification of patients, forecasting tumor responses, and pinpointing new targets for drug discovery. Numerous databases dedicated to cancer research, including the Gene Expression Omnibus (GEO) database, have been established. Identifying differentially expressed genes (DEGs) and key genes deepens our understanding of the initiation of glioblastoma, potentially unveiling novel markers for diagnosis and prognosis, as well as targets for the treatment of glioblastoma.

**Methods:**

This research sought to discover genes implicated in the development and progression of glioblastoma by analyzing microarray datasets GSE13276, GSE14805, and GSE109857 from the GEO database. DEGs were identified, and a function enrichment analysis was performed. Additionally, a protein-protein interaction network (PPI) was constructed, followed by module analysis using the tools STRING and Cytoscape.

**Results:**

The analysis yielded 88 DEGs, consisting of 66 upregulated and 22 downregulated genes. These genes' functions and pathways primarily involved microtubule activity, mitotic cytokinesis, cerebral cortex development, localization of proteins to the kinetochore, and the condensation of chromosomes during mitosis. A group of 27 pivotal genes was pinpointed, with biological process analysis indicating significant enrichment in activities, such as division of the nucleus during mitosis, cell division, maintaining cohesion between sister chromatids, segregation of sister chromatids during mitosis, and cytokinesis. The survival analysis indicated that certain genes, including PCNA clamp-associated factor (PCLAF), ribonucleoside-diphosphate reductase subunit M2 (RRM2), nucleolar and spindle-associated protein 1 (NUSAP1), and kinesin family member 23 (KIF23), could be instrumental in the development, invasion, or recurrence of glioblastoma.

**Conclusion:**

The identification of DEGs and key genes in this study advances our comprehension of the molecular pathways that contribute to the oncogenesis and progression of glioblastoma. This research provides valuable insights into potential diagnostic and therapeutic targets for glioblastoma.

## 1 INTRODUCTION

Glioblastoma is recognized as the most common primary malignancy within the brain, characterized by its relentless invasiveness, diversity, and aggressive behavior, making it notoriously difficult to cure [1]. The emergence and progression of this deadly form of glioma are attributed to abnormal gene expression and a variety of mutations, including those affecting isocitrate dehydrogenase 1 (IDH-1) and isocitrate dehydrogenase 2 (IDH-2), as well as the methylation of O (6)-methylguanine-DNA methyltransferase (MGMT), in addition to mutations in tumor-suppressor genes [2, 3]. As a result, glioblastomas lacking mutations in IDH frequently exhibit elevated levels of epidermal growth factor receptor (EGFR) amplification, alterations in the promoter region of telomerase reverse transcriptase (TERT), and deletion of phosphatase and tensin homolog deleted on chromosome ten (PTEN) [4-7]. Moreover, the presence of MGMT promoter methylation in 30% to 50% of IDH wild-type glioblastomas might be indicative of a somewhat improved prognosis and a beneficial reaction to alkylating chemotherapy agents, such as Temozolomide (TMZ) [8]. However, the notably high mortality associated with glioblastoma can be attributed to the lack of efficient diagnostic methods in the early stages of the disease. Therefore, it is crucial to thoroughly elucidate the molecular processes underlying the oncogenesis of glioblastoma to facilitate the creation of effective diagnostic and therapeutic strategies.

Morphologically, glioblastomas are classified as astrocytic neoplasms and are classified into subtypes of primary and secondary glioblastomas, affecting patients of different age groups, characterized by different signaling pathways, mutations, RNA and protein expression profiles and, consequently, different patient responses to radiо- and chemotherapy. Most glioblastomas develop rapidly de novo from normal glial cells and are common in older patients (primary tumor). Secondary glioblastoma progress from diffuse low-grade astrocytoma or anaplastic astrocytoma, is much less common, predominantly in young patients, in the frontal lobes of the brain, characterized by a lesser degree of necrosis and a more favorable prognosis compared to primary glioblastomas. Although primary and secondary glioblastomas are histologically indistinguishable, their genetic and epigenetic molecular profiles differ [9-14].

In recent years, the application of microarray technologies and bioinformatic analyses for comprehensive genome-wide screening of genetic alterations has played a pivotal role in identifying differentially expressed genes (DEGs) and elucidating the functional pathways implicated in the onset and progression of glioblastoma. However, the potential for high rates of false positives in independent microarray analyses presents significant obstacles to obtaining reliable results. Considering this, the current research entailed accessing and analyzing three mRNA microarray datasets from the Gene Expression Omnibus (GEO) database, aiming to isolate DEGs between glioblastoma and normal brain tissues. This was followed by performing Gene Ontology (GO), Kyoto Encyclopedia of Genes and Genomes (KEGG) pathway analyses, and protein-protein interaction (PPI) network analyses to deepen our understanding of the molecular dynamics at play in cancer genesis and progression. Ultimately, this study successfully identified a total of 88 DEGs and 27 critical genes, offering valuable insights into potential biomarkers for glioblastoma, thereby contributing to the advancement of research in this field.

## 2 MATERIALS AND METHODS

### 2.1 Microarray Data

GEO at http://www.ncbi.nlm.nih.gov/geo serves as a publicly accessible repository for functional genomics data, which includes a vast array of high throughput gene expression data, chips, and microarrays [15]. For this study, three specific gene expression datasets, namely GSE13276, GSE14805, and GSE109857, were retrieved from GEO, all of which were based on the Affymetrix GPL570 platform, utilizing the Affymetrix Human Genome U133 Plus 2.0 Array [16-18]. Within this framework, probe identifiers were mapped to their respective gene symbols using the annotation data provided by the platform. The datasets varied in size: GSE13276 included a collection of 5 tissue samples from patients with glioblastoma alongside 3 samples from non-cancerous brain tissue. GSE14805 comprised a larger dataset containing 34 glioblastoma tissue samples and 4 samples derived from non-cancerous brain tissue. The most extensive dataset, GSE109857, consisted of 89 glioblastoma tissue samples and 5 samples obtained from non-cancerous brain tissue, offering a broad base for comparative analysis. This diverse collection of samples from cancerous and non-cancerous tissues provides a rich resource for examining the differential gene expression profiles associated with glioblastoma, thereby facilitating a deeper understanding of its molecular underpinnings.

### 2.2 Identification of DEGs

The DEGs between glioblastoma and non-cancerous samples were identified using the GEO2R tool, available at http://www.ncbi.nlm.nih.gov/geo/geo2r. GEO2R offers an interactive web-based platform that enables users to compare multiple datasets within a GEO series to pinpoint DEGs across various experimental setups. To strike a balance between identifying statistically significant genes and minimizing the risk of false positives, both *p*-values and the Benjamini and Hochberg false discovery rate were employed. Instances where probe sets did not match any gene symbols or where multiple probe sets corresponded to a single gene were either excluded or the data were averaged, respectively. A threshold for significance was set where a log2-fold change (log2FC) greater than 1 and an adjusted *p*-value of less than 0.001 were deemed significant.

Enrichment analyses for the DEGs were conducted using both the KEGG and GO through the Database for Annotation, Visualization, and Integrated Discovery (DAVID; http://david.ncifcrf.gov) (version 6.7). DAVID serves as an online repository of biological information, amalgamating data, and analytical tools to offer users a comprehensive suite of functional annotations for genes and proteins, facilitating the extraction of valuable biological insights. KEGG stands as a vital resource for understanding the complex functions of biological systems through the integration of large-scale molecular data sets produced by high-throughput experimental methods [19, 20]. Meanwhile, GO represents a crucial bioinformatics instrument for gene and protein annotation, playing a pivotal role in the examination of their biological processes [21]. Functional analysis of the DEGs was executed using the DAVID online database, with a *p*-value of less than 0.05 considered statistically significant, aiming to elucidate the roles of these genes within the pathophysiology of glioblastoma.

### 2.3 PPI Network Construction and Module Analysis

The construction of the PPI network was facilitated by the Search Tool for the Retrieval of Interacting Genes (STRING; http://string-db.org) online database (version 10.0) [22]. Investigating the functional interactions among proteins can shed light on the underlying mechanisms contributing to disease onset and progression. In our study, the STRING database was employed to assemble the PPI network of the identified DEGs, with interactions boasting a combined score greater than 0.4 deemed to carry statistical significance.

For the visualization and further analysis of these PPI networks, Cytoscape (version 3.4.0), a comprehensive and open-source bioinformatics software, was utilized [23]. Within Cytoscape, the Molecular Complex Detection (MCODE) plug-in (version 1.4.2) serves as a powerful application designed to cluster networks based on their topological structures, thereby highlighting densely interconnected regions [24]. The PPI networks were meticulously crafted using Cytoscape, and the principal module within these networks was pinpointed through MCODE. The selection criteria applied for identifying significant modules included an MCODE score exceeding 5, a degree cut-off of 2, a node score cut-off of 0.2, a maximum depth of 100, and a k-score of 2. Following the identification of the key module, both KEGG and GO analyses were conducted on the genes within this module utilizing the DAVID database. This comprehensive approach allows for a deeper understanding of the functional roles and biological pathways of the genes involved in the most significant module of the PPI network.

### 2.4 Hub Genes Selection and Analysis

Hub genes were identified based on their connectivity with degrees of 10 or higher. To explore these genes and their associated co-expression networks, the cBioPortal online platform (http://www.cbioportal.org) was utilized [25, 26]. This analysis facilitated a deeper insight into the interplay and co-expression patterns among these central genes.

For examining the biological processes involving these hub genes, the Biological Networks Gene Ontology tool (BiNGO) plugin for Cytoscape (version 3.0.3) was employed [27]. This tool enabled the effective visualization and analysis of the biological processes, offering a detailed understanding of the roles played by these hub genes within various cellular functions and pathways.

To categorize the hub genes based on their expression patterns and potential relationships, hierarchical clustering was conducted using the UCSC Cancer Genomics Browser (http://genome-cancer.ucsc.edu) [28]. This process helped in organizing the hub genes into clusters based on their expression similarities, providing insights into their potential functional correlations and roles in glioblastoma.

The assessment of the impact of hub genes on overall survival and disease-free survival was conducted through Kaplan-Meier curve analyses within the cBioPortal. These analyses are crucial for understanding how variations in the expression of these genes might influence the prognosis and survival outcomes of glioblastoma patients.

Additionally, the expression profiles of specific hub genes, such as ribonucleoside-diphosphate reductase subunit M2 (RRM2) and kinesin family member 23 (KIF23) were scrutinized and illustrated using the Oncomine database (http://www.oncomine.com). This examination was extended to assess the correlation between the expression patterns of these genes and clinical factors, including initial treatment responses, recurrence status, and the methylation status of MGMT, as well as through the Oncomine platform. This comprehensive approach, utilizing multiple databases and tools, enhances our understanding of the significance and potential impact of hub genes in glioblastoma, offering valuable insights into their roles in tumor biology and clinical outcomes.

## 3 RESULTS

### 3.1 Identification of DEGs in Glioblastoma

**Fig. (1):**
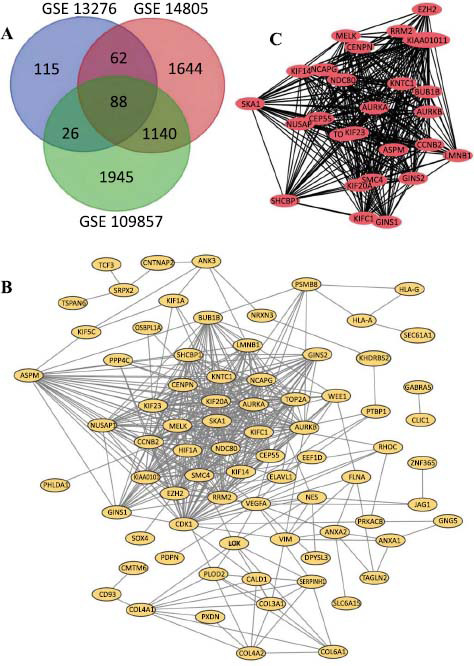
Venn diagram, the protein-protein interaction network (PPI) network and the most significant module of differentially expressed genes (DEGs): (**A**) DEGs were selected with a fold change>2 and *p*-value <0.001 the mRNA expression profiling sets GSE13276, GSE 14805 and GSE 109857. The 3 datasets showed an overlap of 88 genes. (**B**) The PPI network of DEGs was constructed using Cytoscape. (**C**) The most significant module was obtained from the PPI network with 27 hub genes.

Following the normalization of microarray data, a distinct number of DEGs were identified across the datasets: 291 DEGs in GSE13276, 2934 DEGs in GSE14805, and 3199 DEGs in GSE109857 (Fig. **1**). A Venn diagram was employed to illustrate the intersection across these datasets, revealing a commonality of 88 genes. Within this shared subset, 66 genes were found to be upregulated, while 12 genes were downregulated when comparing glioblastoma tissues to non-cancerous brain tissues, as depicted in Fig. (**1A**). This analysis highlights the significant genes that are consistently altered across different studies, providing a focused list of candidates for further investigation into their roles in glioblastoma pathogenesis.

### 3.2 KEGG and GO Enrichment Analyses of DEGs

Functional and pathway enrichment analyses of the DEGs were conducted utilizing the DAVID database to elucidate the biological classifications of these genes. The GO analysis demonstrated that alterations in the biological processes (BP) associated with the DEGs predominantly encompassed areas, such as movement based on microtubules, the process of mitotic cytokinesis, the development of the cerebral cortex, the localization of proteins to the kinetochore, and divisions occurring during mitotic nuclear phases (Table **1**). As for changes in molecular function (MF), the DEGs showed significant enrichment in functions related to the activity of microtubule motors, structural constituents of the extracellular matrix, ATPase activity, and the ability to bind ATP (Table **2**). Regarding changes in cellular components (CC), the DEGs were primarily enriched in the kinesin complex, spindle microtubules, the midbody, and collagen trimers (Table **3**). Additionally, the KEGG pathway analysis indicated that the downregulated DEGs were chiefly enriched in pathways involving focal adhesion, the phosphoinositide 3-kinases (PI3Ks)/protein kinase B (Akt) signaling pathway, and the digestion and absorption of proteins (Table **4**). These findings provide a comprehensive overview of the significant biological processes, molecular functions, and cellular components affected by DEGs in glioblastoma, as well as the key pathways that may be disrupted or altered in the disease state.

### 3.3 PPI Network Construction and Module Analysis

The PPI network pertaining to the DEGs was developed, as illustrated in Fig. (**1B**). Through the application of Cytoscape, a significant module within this network was identified and illustrated in Fig. (**1C**). Subsequent functional analysis of the genes within this crucial module was carried out using the DAVID database. The analysis revealed that the genes constituting this module were predominantly involved in and showed significant enrichment for processes and components, such as focal adhesion, movement facilitated by microtubules, the kinesin complex, and activities associated with microtubule motors, as detailed across Table **1** through **4**. This comprehensive examination underscores the pivotal biological functions and pathways that these module-associated genes partake in, shedding light on their potential roles in the pathophysiology of diseases such as glioblastoma.

### 3.4 Hub Gene Selection and Analysis

A total of 27 genes were pinpointed as key hub genes, each possessing connectivity degrees of 10 or more. Details regarding the names, abbreviations, and functionalities of these pivotal hub genes are presented in Table **5**. Hierarchical clustering analysis demonstrated that these hub genes have the capability to effectively distinguish between glioblastoma samples and non-cancerous counterparts, as depicted in Fig. (**2**).

In the analysis focusing on the BP pathway enrichment of the 27 identified hub genes in glioblastoma, it was found that these genes predominantly contribute to processes such as mitotic nuclear division, cell division, cohesion of sister chromatids, segregation of sister chromatids during mitosis, and mitotic cytokinesis, as outlined in Table **6**. Further, the prognostic significance of these hub genes was evaluated through overall survival analysis using Kaplan-Meier curves. The analysis indicated that glioblastoma patients exhibiting genomic alterations in PCNA clamp-associated factor (PCLAF), RRM2, nucleolar and spindle-associated protein 1 (NUSAP1), and KIF23 displayed reduced overall survival rates, as shown in Fig. (**3**). Moreover, alterations in RRM2 and KIF23 were associated with diminished disease-free survival rates for glioblastoma patients, as illustrated in Fig. (**3**).

Particularly, RRM2 and KIF23, with the highest node degrees of 22, were closely associated with poorer overall survival rates, underscoring their potentially critical roles in glioblastoma oncogenesis. Data derived from cBioPortal revealed that genomic alterations in RRM2 significantly correlated with both overall survival (*p*=3.315e-3) and disease-free survival (*p*=5.423e-3). Conversely, alterations in KIF23 did not show a significant association with either overall survival (*p*=0.281) or disease-free survival (*p*=0.422), as demonstrated in Fig. (**3**).

The expression profiles of RRM2 and KIF23 across various human tissues were examined using the Oncomine database. It was observed that mRNA levels of RRM2 were elevated in a wide range of cancers, including bladder, central nervous system (CNS) tumors, breast, cervical, colorectal, esophageal, gastric, head and neck, kidney, liver, lung, lymphoma, melanoma, ovarian, pancreatic, and sarcoma when compared to their normal tissue counterparts, as highlighted in Fig. (**4A**). Similarly, KIF23 mRNA levels were found to be higher in cancers, such as bladder, CNS tumors, breast, cervical, colorectal, esophageal, gastric, head and neck, liver, lung, lymphoma, melanoma, ovarian, pancreatic, and sarcoma compared to normal tissues, as depicted in Fig. (**4B**). Oncomine's comparative analysis between cancer and normal tissues revealed that both RRM2 and KIF23 were significantly overexpressed in glioma across various datasets, showcased in Fig. (**5**). Furthermore, in these datasets, elevated mRNA levels of RRM2 and KIF23 were linked with parameters, such as initial treatment response, recurrence status, and MGMT Methylation status, as illustrated in Fig. (**6**).

## 4 DISCUSSION

Glioblastoma stands as the most frequently diagnosed aggressive brain tumor, accounting for about 57% of all cases of gliomas [29]. The treatment approach for glioblastoma is multi-faceted, encompassing surgical intervention, radiation therapy, systemic treatments (which include chemotherapy and targeted therapies), along with supportive care measures. Despite these comprehensive treatment strategies, the overall outlook for patients with glioblastoma continues to be bleak, with long-term survival being an uncommon outcome. This grim prognosis is largely attributed to the highly malignant nature of glioblastoma, characterized by its rapid growth, invasive behavior, and significant heterogeneity within the tumor, both genetically and phenotypically. Such complexity not only challenges the efficacy of conventional therapies but also contributes to the high recurrence rate after initial treatment success. The aggressive nature of glioblastoma, coupled with its capacity to extensively infiltrate brain tissue, often renders complete surgical resection impossible, leaving residual tumor cells that contribute to relapse.

Furthermore, the blood-brain barrier poses a significant obstacle to many systemic therapies, limiting the effectiveness of chemotherapeutic agents and targeted therapies in reaching the tumor site at therapeutic concentrations [30, 31]. Considering these challenges, research efforts continue to focus on understanding the underlying molecular mechanisms driving glioblastoma pathogenesis and resistance to therapy, with the goal of identifying novel therapeutic targets and improving treatment strategies [32]. However, until significant breakthroughs are achieved, the management of glioblastoma remains a daunting task, emphasizing the urgent need for continued research and innovation in the field of neuro-oncology.

The identification of risk factors for glioblastoma development has been limited. Among these, exposure to ionizing radiation stands out as the most significant risk factor linked to glioblastoma genesis, representing the sole risk factor that could potentially be modified [33]. Additionally, there has been an observed inverse relationship between the incidence of glioblastoma and the presence of atopy, allergies, and various immune-related conditions, though the specific biological mechanisms underlying this association remain unclear [34, 35]. While certain rare genetic conditions, such as Li-Fraumeni syndrome and Lynch syndrome, have been associated with an increased risk of glioblastoma, they contribute to less than 1% of the total cases [36]. A notable challenge in glioblastoma management is that most cases are diagnosed at an advanced stage, at which point curative treatment options are not viable, contributing to the overall poor prognosis for these patients. Consequently, there is a pressing need for the identification of effective diagnostic and therapeutic markers. The use of microarray technology has emerged as a powerful tool in the exploration of genetic changes in glioblastoma, offering promise for the discovery of novel biomarkers that could pave the way for advancements in the diagnosis and treatment of this disease and potentially other conditions.

In the last decade, along with the development of high-throughput technologies, such as microarrays and next-generation sequencing (NGS) and microarray technologies, multi-omics data have been obtained, for example, genomics, transcriptomics, proteomics, and metabolomics data [37, 38]. These multi-omics data provide enormous useful information for improving tumor therapy strategies. First, compared to searching for new compounds for anticancer therapy, identifying new approaches to using existing drugs is much more cost-effective. Generation of multi-omics data enables computational prediction of anticancer drugs based on drug repositioning. Drug repositioning, which concerns the discovery and development of new clinical indications for existing drugs or those in development, has become an increasingly important strategy for new drug discovery. Secondly, one of the biggest problems in modern glioma therapy is that tumors are heterogeneous [39]. Over the past 50 years, there have been numerous studies showing that patients with gliomas have very different responses to treatment with the same drug. Understanding a patient's specific genetic, epigenetic, and transcriptomic profile is useful for understanding the mechanisms of tumorigenesis, with the potential to find effective diagnostic, prognostic, and therapeutic tools. With the development of NGS/microarray technologies, it is now possible to obtain multi-omics information for patients with gliomas before determining a specific treatment regimen. In this decade, big data methods have been widely used to interpret NGS/microarray data, search for new biomarkers, predict drug sensitivity (or resistance), and finally, for precise anticancer drug selection. In this context, researchers are taking advantage of genomics, bioinformatics, and molecular biology tools to find molecules that provide valuable tumor information and clinical relevance [40-44]. This study was designed to deliver new insights into glioblastoma, which is a multi-gene hereditary disease, while also exploring promising novel biomarkers for diagnosis, prognosis, and targeted therapies.

In this study, analysis was conducted on three mRNA microarray datasets to identify DEGs between glioma-cancerous tissues and their non-cancerous counterparts. From this analysis, a collective total of 88 DEGs were pinpointed across all datasets. Subsequent GO and KEGG enrichment analyses were carried out to delve into the interactions and functions of these DEGs. The findings from the GO analysis indicated that alterations in the BP of the DEGs were notably concentrated in areas, such as movement driven by microtubules, the process of cytokinesis during mitosis, the development of the cerebral cortex, the localization of proteins to the kinetochore, and the division of cells during mitosis. In terms of MF, there was a significant enrichment in activities related to the functioning of microtubule motors, components of the extracellular matrix, ATPase activity, and ATP binding capabilities. The alterations in the CC aspect of DEGs showed a significant presence in structures such as the kinesin complex, microtubules associated with the spindle, the midbody of dividing cells, and the collagen trimer. From the KEGG pathway analysis, it was discovered that the genes that were downregulated among the DEGs predominantly participated in pathways related to focal adhesion, the signaling pathway involving PI3Ks/Akt, and the digestion and absorption of proteins.

In this investigation, 27 DEGs were identified as central hub genes, each exhibiting connectivity degrees of 10 or more. Notably, RRM2 and KIF23 emerged as the genes with the highest connectivity, both displaying node degrees of 22 and were significantly correlated with reduced overall survival rates. RRM2, serving as the smaller subunit of ribonucleotide reductase, has been acknowledged as both a promoter of tumor growth and a viable target for cancer therapy [45]. The prevalence of RRM2's high expression across various cancers further solidifies its role as a facilitator of tumorigenesis and a strategic point of intervention for cancer treatment [46]. RRM2 has been targeted by anticancer agents, notably, the antisense oligonucleotide GTI2040, which has demonstrated substantial antitumor efficacy in preclinical models and has undergone phase I clinical trials [47].

Additionally, RRM2's overexpression has been linked to the emergence of resistance against a spectrum of chemotherapeutic agents, including Gemcitabine, Doxorubicin (DOX), and Imatinib, among others [48-52]. This overexpression is also prevalent in various cancers that exhibit chemotherapy resistance, where RRM2's upregulation activates complex signaling pathways that regulate survival, growth, apoptosis, and chemoresistance in cancer cells. Consequently, targeting RRM2 presents a promising strategy for counteracting chemoresistance by promoting apoptosis, inhibiting cell growth, and disrupting the processes of DNA replication and repair.

Within the context of this study, the PPI network analysis revealed direct interactions between RRM2 and several key proteins including spindle and kinetochore-associated complex subunit 1 (SKA1), SHC binding, and spindle-associated 1 (SHCBP1), structural maintenance of chromosomes protein 4 (SMC-4), WEE1, vascular endothelial growth factor A (VEGF-A), and DNA topoisomerase 2-alpha (TOP2A). These interactions underscore the pivotal role that RRM2 plays in the pathology of malignant gliomas, highlighting its importance not only as a marker of poor prognosis but also as a critical target for therapeutic intervention.

Amplification of the human RRM2 gene, originating from a region of homogeneous staining on chromosomes, is consistently linked with changes in transcriptional regulation. Clones resistant to Gemcitabine and Hydroxyurea have demonstrated distinct interaction patterns with a variety of transcription factors, such as activator protein 1 (AP-1), Sp1, cAMP-response element-binding protein (CREB), and nuclear factor kappa-B (NF-kB) [53]. Subsequent studies uncovered that Vasohibin 2 (VASH2) could stimulate AP-1, leading to its binding to the RRM2 promoter, thereby enhancing the transactivation of RRM2, a mechanism correlated with resistance to Gemcitabine [54]. Additionally, the activation of checkpoint kinase 1 (Chk1) in response to DNA damage has been shown to elevate RRM2 expression *via* the E2F1 transcription factor [55]. Consequently, various compounds, including inhibitors of cyclin-dependent kinases, Src tyrosine kinases, and gambogic acid, have been observed to augment Gemcitabine's efficacy by diminishing RRM2 expression through the inhibition of E2F1 [34, 56, 57]. Moreover, the transcriptional upregulation of nuclear factor Y (NF-Y), triggered by histone deacetylase (HDAC) inhibition, has been identified to increase RRM2 transcription, thereby playing a role in the development of resistance to Gemcitabine [58].

**Fig. (7):**
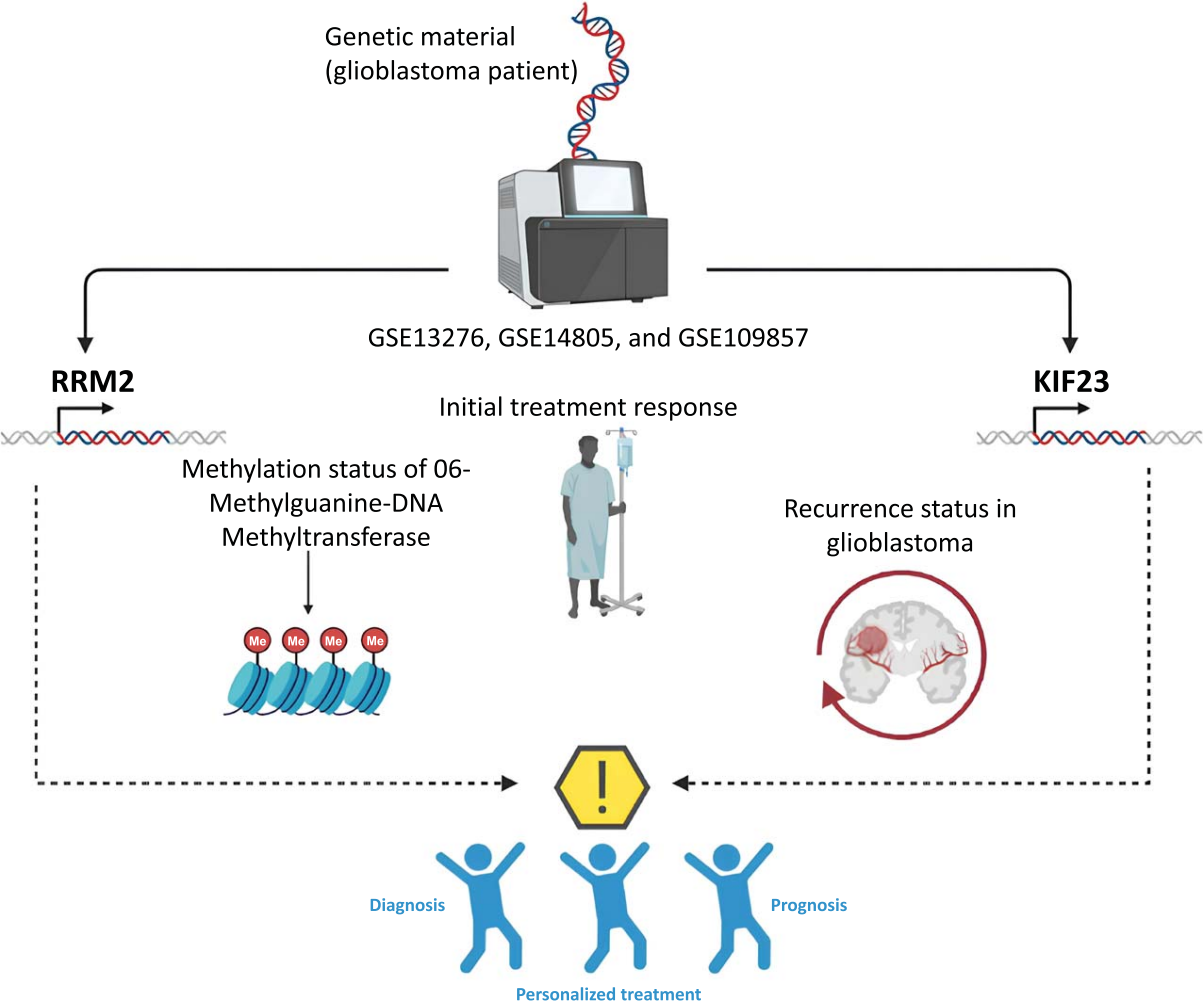
Clinical potential of ribonucleoside-diphosphate reductase subunit M2 (RRM2) and kinesin family member 23 (KIF23) in relation to glioblastoma.

This study posits that the analysis of survival in cBioPortal, which hinges on the correlation between gene mutations and prognostic outcomes, might overlook instances where gene overexpression stems from amplification rather than mutation. Hence, the overexpression of RRM2 in glioblastoma could predominantly result from gene amplification, a hypothesis that warrants further investigation for validation. Analysis utilizing Oncomine indicated that RRM2 and KIF23 expression patterns are significantly associated with the initial treatment response, the methylation status of MGMT, and the recurrence status in glioblastoma, suggesting their potential as markers for treatment strategy and prognosis evaluation (Fig. **7**).

KIF23, the human counterpart of the mouse's KIF23, functions as a nuclear protein that predominantly localizes to the interzone of mitotic spindles, operating as a motor enzyme that directs movement along microtubules in an antiparallel fashion *in vitro* [59-63]. It has been documented in previous research that the reduction of KIF23 levels in HeLa cells results in the emergence of multinucleated cells, a phenomenon attributed to disruptions in cytokinesis [64, 65]. Microtubules, which serve as the pathways for KIF23's motor activity, play crucial roles in determining cell shape and facilitating processes, such as cell motility, mitosis, the transport of intracellular vesicles, and the spatial arrangement of membranes and organelles within the cell [63-70]. Given their central role in these critical cellular functions, microtubules have been targeted in the development of various cancer chemotherapies [64]. Considering the function of KIF23 in microtubule dynamics, its elevated expression levels in gliomas relative to normal brain tissue, and its identification through SEREX as a potential novel antigen for glioma, it is hypothesized that KIF23 may play a significant role in the development of glioblastoma [71, 72].

In the biomarker/therapeutic agent discovery phase, bioinformatics methods have gained relevance due to their ability to identify DEGs, proteins, and peptides in healthy and diseased tissues with the goal of characterizing which of these expressed molecules could potentially be biomarker or therapeutic targets for a particular tumor. Storing information in databases such as GEO has facilitated computational bioprospecting [73, 74]. However, it is important to recognize that this study is based on *in silico* analysis, and although the results are theoretically sound, they have not yet been confirmed experimentally. Although the use of an external validation dataset in the present study may serve as a somewhat viable alternative to experimental validation, we hypothesize that experimental study and cell and animal model validation would be a more robust approach. In future studies, we may combine gene expression levels of RRM2 and KIF23 and cell biological behavior analysis to further explore the oncogenesis of glioblastoma.

## CONCLUSION

To summarize, this study aimed to discover DEGs that could play roles in the onset or advancement of glioblastoma. Through our analysis, we identified 88 DEGs and pinpointed 27 central hub genes, which hold the potential to serve as diagnostic markers for glioblastoma. Nevertheless, additional research is required to comprehensively understand the specific roles and biological functions these genes may have in the context of glioblastoma. Such an in-depth exploration would not only contribute to a more nuanced understanding of glioblastoma's molecular underpinnings but also pave the way for the development of targeted therapies. The identification of these genes as potential biomarkers offers a promising avenue for early detection and personalized treatment strategies, which are crucial in improving the prognosis and quality of life for patients with glioblastoma. By further investigating these genes, researchers can potentially uncover novel therapeutic targets, thereby expanding the arsenal against this aggressive form of CNS tumor. Ultimately, the integration of genetic insights with clinical strategies holds the key to advancing the field of glioblastoma treatment and research.

## Figures and Tables

**Fig. (2):**
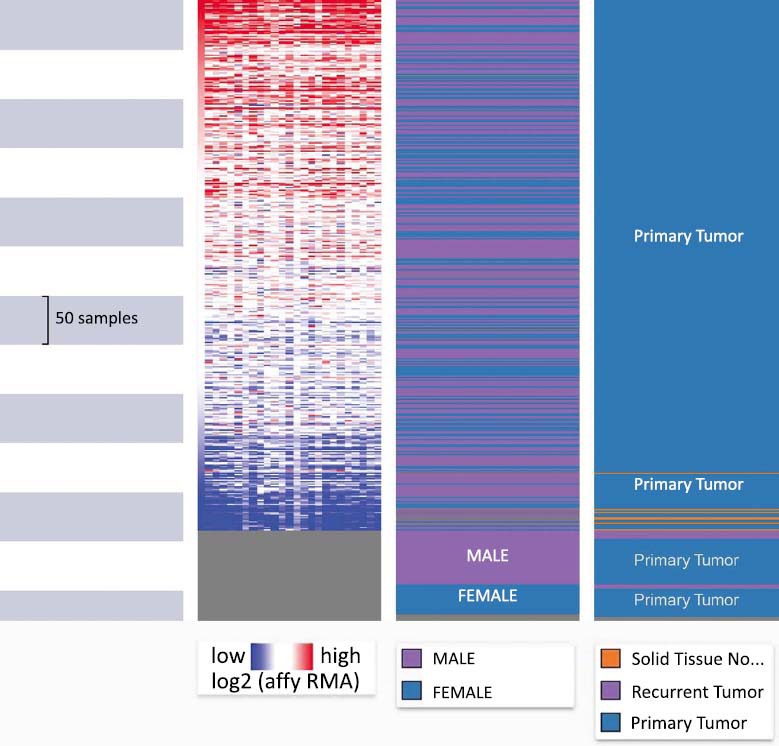
Hierarchical clustering of hub genes was constructed using the UCSC Cancer Browser.

**Fig. (3):**
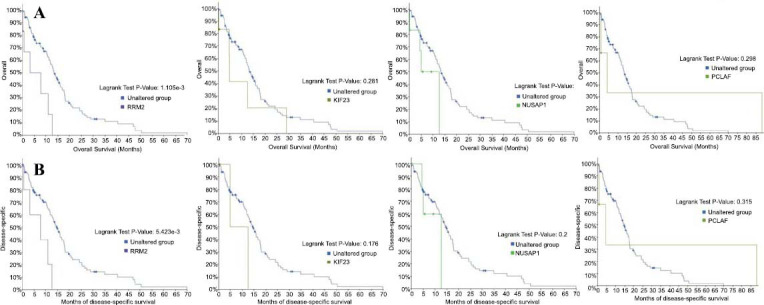
(**A**) Overall survival and (**B**) disease-free survival analyses of hub genes were performed using the cBioportal online platform. *p* <0.05 and considered statistically significant.

**Fig. (4):**
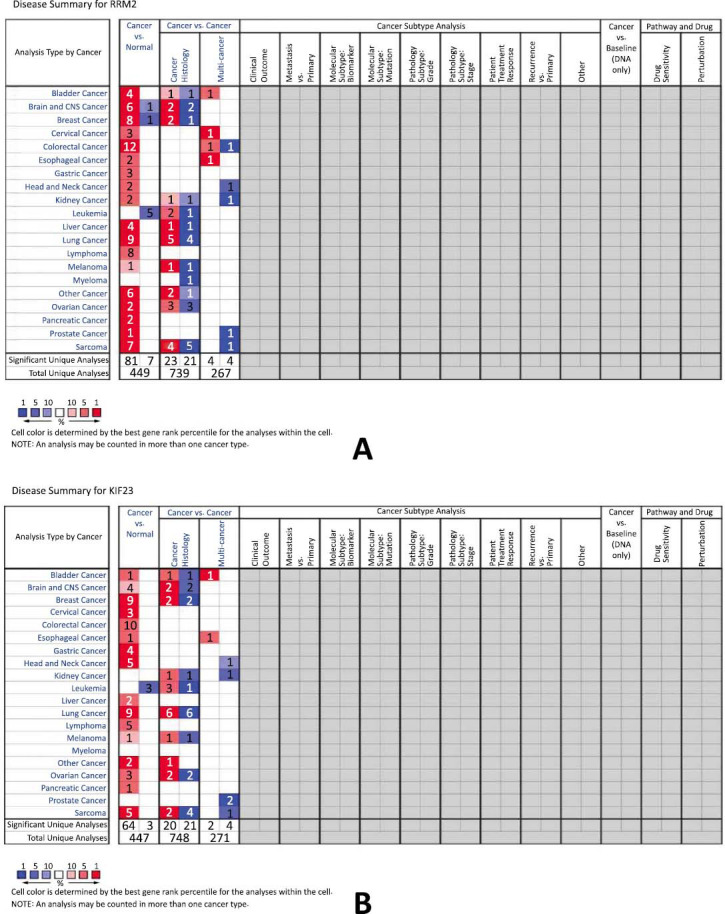
(**A**) and (**B**) oncomine analysis of ribonucleoside-diphosphate reductase subunit M2 (RRM2) and kinesin family member 23 (KIF23) genes expression in different cancers.

**Fig. (5):**
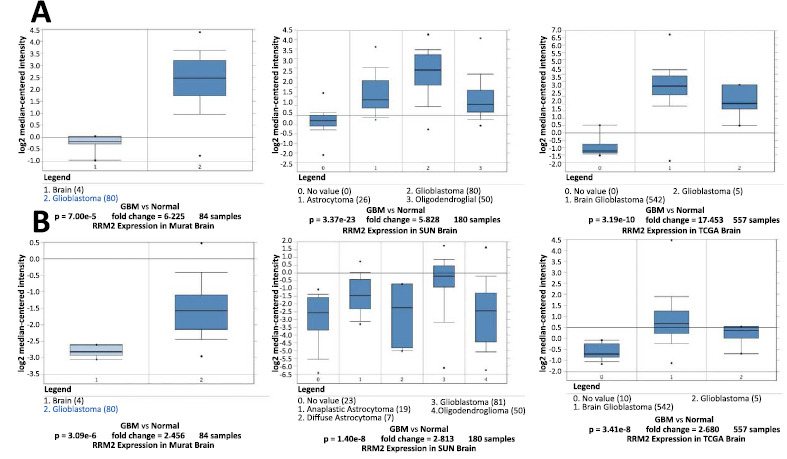
(**A**) and (**B**) oncomine analysis of cancer *vs.* normal tissue showed that ribonucleoside-diphosphate reductase subunit M2 (RRM2) and kinesin family member 23 (KIF23) were significantly overexpressed in glioma in the different datasets.

**Fig. (6):**
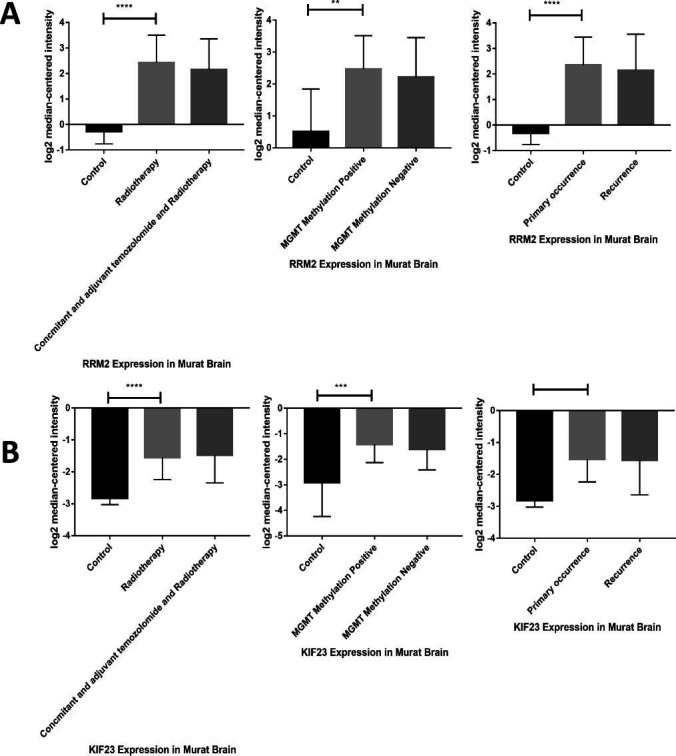
Higher mRNA levels of ribonucleoside-diphosphate reductase subunit M2 (RRM2) (**A**) and kinesin family member 23 (KIF23) (**B**) were associated with initial treatment, recurrence status, and O (6)-methylguanine-DNA-methyltransferase (MGMT) Methylation status.

**Table 1:** Biological processes (BP) pathway enrichment analysis of differentially expressed genes (DEGs) in glioblastoma.

**Term**	**Description**	**Count**	***p*-Value**
GO:0007018	Microtubule-based movement	6	3.16E-05
GO:0000281	Mitotic cytokinesis	4	2.46E-04
GO:0021987	Cerebral cortex development	4	0.001139829
GO:0034501	Protein localization to kinetochore	3	0.00151097
GO:0007067	Mitotic nuclear division	4	0.001564823
GO:0007076	Mitotic chromosome condensation	3	0.001839669
GO:0030705	Cytoskeleton-dependent intracellular transport	3	0.003458665
GO:0007052	Mitotic spindle organization	3	0.007408114
GO:0032467	Positive regulation of cytokinesis	3	0.007408114
GO:0030199	Collagen fibril organization	3	0.012677496
GO:0051272	Positive regulation of cellular component movement	2	0.017670854

**Table 2:** Molecular function (MF) pathway enrichment analysis of differentially expressed genes (DEGs) in glioblastoma.

**Term**	**Pathway Description**	**Count**	***p*-Value**
GO:0005871	Kinesin complex	6	4.77E-06
GO:0005876	Spindle microtubule	5	2.36E-05
GO:0030496	Midbody	6	6.53E-05
GO:0005581	Collagen trimer	5	7.07E-05
GO:0005874	Microtubule	5	0.002346848
GO:0051233	Spindle midzone	3	0.003188572
GO:0016328	Lateral plasma membrane	3	0.013235985
GO:0000796	Condensin complex	2	0.021099162
GO:0042383	Sarcolemma	3	0.022842198
GO:0005737	Cytoplasm	22	0.022951589

**Table 3:** Changes in cell component (CC) pathway enrichment analysis of differentially expressed genes (DEGs) in glioblastoma.

**Term**	**Description**	**Count in Gene Set**	***p*-value**
cfa04510:	Focal adhesion	6	0.003963
cfa04151:	PI3K-Akt signaling pathway	7	0.007034
cfa04974:	Protein digestion and absorption	4	0.009134
cfa04512:	ECM-receptor interaction	4	0.010054
cfa05146:	Amoebiasis	4	0.017115
cfa05205:	Proteoglycans in cancer	5	0.017563
cfa04114:	Oocyte meiosis	4	0.017985
cfa04110:	Cell cycle	4	0.025292
cfa05206:	MicroRNAs in cancer	4	0.034612
cfa04115:	p53 signaling pathway	3	0.044317
cfa05200:	Pathways in cancer	6	0.048794

**Table 4:** Kyoto Encyclopedia of Genes and Genomes (KEGG) pathway enrichment analysis of differentially expressed genes (DEGs) in glioblastoma.

**Term**	**Pathway Description**	**Count**	***p*-value**
GO:0003777	Microtubule motor activity	5	2.36E-04
GO:0005201	Extracellular matrix structural constituent	4	9.42E-04
GO:0016887	ATPase activity	5	0.001533
GO:0005524	ATP binding	14	0.005736
GO:0035174	Histone serine kinase activity	2	0.025983
GO:0004871	Signal transducer activity	4	0.026153
GO:0005200	Structural constituent of cytoskeleton	3	0.028268
GO:0004859	Phospholipase inhibitor activity	2	0.031099

**Table 5:** Functional roles of 27 hub genes with degree-10.

**Gene Symbol**	**Full Name**	**Function**
GINS1	DNA replication complex GINS protein PSF1	The GINS complex plays an essential role in the initiation of DNA replication, and progression of DNA replication forks.
SKA1	Spindle and kinetochore-associated protein 1	Component of the SKA1 complex, a microtubule-binding subcomplex of the outer kinetochore that is essential for proper chromosome segregation.
BUB1B	Mitotic checkpoint serine/threonine-protein kinase BUB1 beta	Essential component of the mitotic checkpoint. Required for normal mitosis progression.
CCNB2	G2/mitotic-specific cyclin-B2	CCNB2 (cyclin B2) is associated with invasion, metastasis and poor prognosis of several cancers.
MELK	Maternal embryonic leucine zipper kinase	Serine/threonine-protein kinase involved in various processes such as cell cycle regulation, self-renewal of stem cells, apoptosis and splicing regulation.
KIAA0101	PCNA-associated factor	PCNA-binding protein that acts as a regulator of DNA repair during DNA replication.
SHCBP1	SHC SH2 domain-binding protein 1	May play a role in signaling pathways governing cellular proliferation, cell growth and differentiation.
AURKB	Aurora kinase B	Serine/threonine-protein kinase component of the chromosomal passenger complex (CPC), a complex that acts as a key regulator of mitosis.
EZH2	Histone-lysine N-methyltransferase EZH2	Catalytic subunit of the PRC2/EED-EZH2 complex, which methylates 'Lys-9' (H3K9me) and 'Lys- 27' (H3K27me) of histone H3, leading to transcriptional repression of the affected target gene.
KNTC1	Kinetochore-associated protein 1	Essential component of the mitotic checkpoint, which prevents cells from prematurely exiting mitosis.
SMC4	Structural maintenance of chromosomes protein 4	Central component of the condensin complex, a complex required for conversion of interphase chromatin into mitotic-like condense chromosomes.
RRM2	Ribonucleoside-diphosphate reductase subunit M2	Provides the precursors necessary for DNA synthesis.
KIF14	Kinesin-like protein KIF14	Microtubule motor protein that binds to microtubules with high affinity through each tubulin heterodimer and has an ATPase activity (By similarity).
LMNB1	Lamin-B1	Lamins are components of the nuclear lamina, a fibrous layer on the nucleoplasmic side of the inner nuclear membrane, which is thought to provide a framework for the nuclear envelope and may also interact with chromatin.
CENPN	Centromere protein N	Component of the CENPA-NAC (nucleosome-associated) complex, a complex that plays a central role in assembly of kinetochore proteins, mitotic progression and chromosome segregation.
KIFC1	Kinesin-like protein KIFC1	Minus end-directed microtubule-dependent motor required for bipolar spindle formation.
KIF20A	Kinesin-like protein KIF20A	Mitotic kinesin required for chromosome passenger complex (CPC)-mediated cytokinesis.
CEP55	Centrosomal protein of 55 kDa	Plays a role in mitotic exit and cytokinesis.
TOP2A	Topoisomerase(DNA)II ɑ	Control of topological states of DNA by transient breakage and subsequent rejoining of DNA strands.
NUSAP1	Nucleolar and spindle associated protein 1	High expression of NUSAP1 is involved in the progression of prostate cancer.
CDK1	Cyclin-dependent kinase 1	Plays a key role in the control of the eukaryotic cell cycle by modulating the centrosome cycle as well as mitotic onset.
ASPM	Abnormal spindle-like microcephaly-associated protein	The function in regulating microtubule dynamics at spindle poles including spindle orientation, astral microtubule density and poleward microtubule flux seems to depend on the association with the katanin complex formed by KATNA1 and KATNB1.
AURKA	Aurora kinase A	Mitotic serine/threonine kinase that contributes to the regulation of cell cycle progression.
NCAPG	Activation of CDK1 at centrosomes	Regulatory subunit of the condensin complex, a complex required for conversion of interphase chromatin into mitotic-like condense chromosomes.
GINS2	DNA replication complex GINS protein PSF2	The GINS complex plays an essential role in the initiation of DNA replication, and progression of DNA replication forks.
KIF23	Kinesin-like protein KIF23	Component of the centralspindlin complex that serves as a microtubule-dependent and Rho-mediated signaling required for the myosin contractile ring formation during the cell cycle cytokinesis.
NDC80	Kinetochore protein NDC80 homolog	Acts as a component of the essential kinetochore- associated NDC80 complex, which is required for chromosome segregation and spindle checkpoint activity.

**Table 6:** Biological processes (BP) pathway enrichment analysis of hub genes in glioblastoma.

**Term**	**Description**	**Count**	***P*-value**
GO:0007067	Mitotic nuclear division	11	1.11E-12
GO:0051301	Cell division	11	3.37E-11
GO:0007062	Sister chromatid cohesion	6	3.79E-07
GO:0000070	Mitotic sister chromatid segregation	4	6.56E-06
GO:0000281	Mitotic cytokinesis	4	1.04E-05
GO:0034501	Protein localization to kinetochore	3	9.51E-05
GO:0007059	Chromosome segregation	4	1.37E-04
GO:0031145	Anaphase-promoting complex-dependent catabolic process	4	2.14E-04
GO:0007076	Mitotic chromosome condensation	3	2.21E-04
GO:0007018	Microtubule-based movement	4	2.30E-04

## Data Availability

All relevant raw data are freely available to any researchers who wish to use them for non-commercial purposes while preserving any necessary confidentiality and anonymity. The datasets are available on request to the corresponding author.

## LIST OF ABBREVIATIONS

GEO: Gene Expression Omnibus
DEGs: Differentially Expressed Genes
NUSAP1: Nucleolar and Spindle-associated Protein 1
IDH-1: Isocitrate Dehydrogenase 1
TERT: Telomerase Reverse Rranscriptase
TMZ: Temozolomide
GO: Gene Ontology
KEGG: Kyoto Encyclopedia of Genes and Genomes
